# Recent social conditions affect boldness repeatability in individual sticklebacks

**DOI:** 10.1016/j.anbehav.2015.12.010

**Published:** 2016-02

**Authors:** Jolle Wolter Jolles, Benjamin Aaron Taylor, Andrea Manica

**Affiliations:** Department of Zoology, University of Cambridge, Cambridge, U.K.

**Keywords:** animal personality, boldness, consistency, housing, isolation, repeatability, three-spined stickleback

## Abstract

Animal personalities are ubiquitous across the animal kingdom and have been shown both to influence individual behaviour in the social context and to be affected by it. However, little attention has been paid to possible carryover effects of social conditions on personality expression, especially when individuals are alone. Here we investigated how the recent social context affected the boldness and repeatability of three-spined sticklebacks, *Gasterosteus aculeatus*, during individual assays. We housed fish either solitarily, solitarily part of the time or socially in groups of four, and subjected them twice to a risk-taking task. The social conditions had a large effect on boldness repeatability, with fish housed solitarily before the trials showing much higher behavioural repeatability than fish housed socially, for which repeatability was not significant. Social conditions also had a temporal effect on the boldness of the fish, with only fish housed solitarily taking more risks during the first than the second trial. These results show that recent social conditions can thus affect the short-term repeatability of behaviour and obfuscate the expression of personality even in later contexts when individuals are alone. This finding highlights the need to consider social housing conditions when designing personality studies and emphasizes the important link between animal personality and the social context by showing the potential role of social carryover effects.

## Highlights

• The role of recent social conditions on boldness repeatability is tested in fish.
• Social isolation before individual trials had a positive effect on repeatability.
• Recent short-term experience of a social group reduced behavioural repeatability.
• Temporal effects resulting from isolation require careful repeatability analysis.
• Social housing conditions need to be evaluated when designing personality research.

It is now well known that animal personalities are omnipresent in the animal kingdom (Réale et al., 2010, Réale et al., 2007, Sih et al., 2004). These consistent individual differences in behaviour play a fundamental role in the social organization of animals (Aplin et al., 2013, Croft et al., 2009, Pike et al., 2008, Sih et al., 2012, Webster and Ward, 2011) and have considerable impact on a range of evolutionary and ecological processes (Réale et al., 2010, Réale et al., 2007, Sih et al., 2012, Smith and Blumstein, 2008, Wolf and Weissing, 2012). However, while the number of studies that document the existence of animal personalities continues to grow (Réale et al., 2007, Sih et al., 2012), there is still a lack of knowledge about the stability of personality traits and the factors that may affect it (Bell and Stamps, 2004, Dingemanse et al., 2010, Laskowski and Pruitt, 2014).

The social environment is one of the major modulating factors of individual behaviour (Van den Bos et al., 2013, Webster and Ward, 2011), and may both restrict and enhance individuals' behavioural responses (Webster & Ward, 2011). For example, individual fish are more active and exploratory in a social group (Gómez-Laplaza and Morgan, 1986, Jolles et al., 2014, Webster et al., 2007), but more persistent in their attention when alone (Gómez-Laplaza & Morgan, 1986). Personality differences affect individual behaviour in a social context, such as risk-taking behaviour (Jolles et al., 2014, Magnhagen and Bunnefeld, 2009), leadership (Harcourt et al., 2009, Jolles et al., 2014, Kurvers et al., 2009), producer-scrounger dynamics (Dyer et al., 2009, Jolles et al., 2013, Kurvers et al., 2010) and the social organization of individuals (Aplin et al., 2013, Croft et al., 2009, Pike et al., 2008). However, the behaviour and personality of individuals are also strongly affected by the social context (Webster & Ward, 2011), and individuals often behave rather plastically across social contexts (David et al., 2011, Morand-Ferron et al., 2011, Van Oers et al., 2005, Webster et al., 2007). Individuals thereby modulate their behaviour based on that of others (Herbert-Read et al., 2012, Reebs, 2000, Webster and Ward, 2011), such as that related to the composition of the group (Magnhagen & Staffan, 2005) and the sex (Piyapong et al., 2010, Schuett and Dall, 2009) and personality (Jolles et al., 2015, Magnhagen and Bunnefeld, 2009, Van Oers et al., 2005) of their group mates. For example, although in three-spined sticklebacks, *Gasterosteus aculeatus*, risk-taking behaviour and leadership of individuals in a social context are positively linked to their propensity to take risks when alone (‘boldness’), this effect can be strongly enhanced or reduced by the personality of their current (Harcourt et al., 2009, Jolles et al., 2015) and previous group mates (Jolles et al., 2014). Consequently, in a social group, the behavioural variance among individuals tends to be reduced (Gómez-Laplaza and Morgan, 1986, Herbert-Read et al., 2012, Magnhagen and Bunnefeld, 2009) and the personalities of individuals, quantified in individual assays, only expressed to a certain extent (Castanheira et al., 2013, Magnhagen and Bunnefeld, 2009, Webster et al., 2007). However, in relatively stable social environments individuals are more likely to repeat certain behaviours by positive feedback from experience and optimal behaviour via repeated interactions (Harcourt et al., 2009, Laskowski and Pruitt, 2014, Nakayama et al., 2013). These interactions may increase the behavioural variability among individuals (Laskowski & Pruitt, 2014) and the behavioural repeatability of individuals (Laskowski and Bell, 2013, Wolf et al., 2011).

If the effect of the social context is so strong, could it be that it still affects the subsequent expression of personality (and thus its repeatability) when individuals are alone? This carryover effect may be likely, as the prior social context has already been shown to affect behaviour in later social contexts in terms of an individual's shoaling decisions (Gómez-Laplaza, 2009), risk-taking behaviour (Frost et al., 2007, Jolles et al., 2014) and leadership (Jolles et al., 2014). Furthermore, it takes time for individuals to adjust between (social) environments, resulting in habituation (decline in behaviour) and/or acclimatization (change in behaviour) responses (Biro, 2012, Budaev, 1997, Gómez-Laplaza and Morgan, 2000, Martin and Réale, 2008), such as individuals becoming less active over solitary test trials (Martin & Réale, 2008) and showing more stable behavioural patterns after longer social isolation (Biro, 2012). Behavioural repeatability may be further compromised at the group level by the large variability in the way individuals are affected by prior social experiences (Jolles et al., 2014), and the speed (Rodríguez-Prieto, Martín, & Fernández-Juricic, 2011) and extent to which they adjust to environmental change (Dingemanse & Wolf, 2013). For example, shy individuals are less affected by previous social experiences than bold individuals (Jolles et al., 2014) and show higher behavioural plasticity between social contexts, in three-spined sticklebacks, perch, *Esox lucius*, and zebra finches, *Taeniopygia guttata* (Jolles et al., 2014, Magnhagen and Bunnefeld, 2009, Magnhagen and Staffan, 2005, Schuett and Dall, 2009, Webster et al., 2007).

Here we investigated to what extent recent social conditions affect the boldness and repeatability of individual three-spined sticklebacks that were either solitarily housed, solitarily housed part of the time or socially housed in small groups of four prior to two trials of a boldness test (see Table 1). As only fish in the solitary treatment had time to habituate and acclimatize to being alone, we hypothesized that these fish would show the most risk-taking behaviour due to lower stress of isolation. We also hypothesized that solitary fish would show the highest repeatability in their behaviour as they had more time for social modulation effects to fade and individual variability in acclimatization responses to stabilize. Fish that were housed solitarily only part of the time were predicted to show intermediate levels of repeatability. We assessed behavioural repeatability by three of the most used indices to get a full picture of personality expression following Bell, Hankison, and Laskowski (2009): agreement repeatability, the extent to which individual differences in trait scores are maintained over time relative to the change of the group (Biro & Stamps, 2015), consistency repeatability, which measures the agreement in relative measurements between individuals (Nakagawa & Schielzeth, 2010), and raw rank order consistency. The three-spined stickleback is an excellent model system to investigate these questions on personality and social dynamics (see e.g. Bell and Sih, 2007, Bell and Stamps, 2004, Harcourt et al., 2009, Jolles et al., 2014, Jolles et al., 2015, Laskowski and Bell, 2014, Pike et al., 2008, Ward et al., 2005, Webster et al., 2007, Webster et al., 2009), as it is a social species, with a strong tendency to shoal most of the year (Huntingford and Coyle, 2010, Ostlund-Nilsson et al., 2010), and is also physically and behaviourally robust, and can thus be kept both solitarily and in groups in a laboratory environment (Huntingford & Ruiz-Gomez, 2009).

## Methods

### Subjects and Housing

We collected three-spined sticklebacks using a sweep net from a tributary of the river Cam, near Cambridge, U.K., and housed them in an environmentally controlled laboratory for at least 4 months before the start of experiments. Ambient temperature was maintained at 14 °C and the photoperiod at 12:12 h light:dark. Fish were kept socially (ca. 200 fish) in a large glass holding aquarium (120 × 60 cm and 60 cm high) with artificial plants, aeration and under-gravel filtration, and fed frozen bloodworms (chironomid larvae) ad libitum once daily. During the experimental period, fish were housed in custom holding tanks (60 × 30 cm and 40 cm high) lined with gravel and divided lengthwise into six compartments (30 × 12 cm and 15 cm depth) by opaque acrylic partitions. Of each tank, five compartments were used to house fish and contained an artificial plant; the remaining compartment contained an under-gravel filter. The partitions prevented fish from seeing conspecifics in adjacent compartments and minimized the transfer of olfactory cues. All fish were of similar length (41 ± 0.7 mm) and age (ca. 12 months) and were taken from a single population to minimize population-specific effects that may influence personality (Bell, 2005). The temperature and photoperiod regime in the laboratory resemble early spring/late autumn conditions, and prevented the fish from coming into breeding condition (Borg et al., 2004, Ostlund-Nilsson et al., 2010). Therefore the sex of the fish was not determined. Fish had not been used in any previous experiments.

### Boldness Test

To investigate an individual's propensity to take risks (‘boldness’), we subjected them individually to one of eight identical white acrylic tanks (70 × 15 cm and 30 cm high) that contained gravel sloping from a deep area (14 cm depth) to an increasingly shallow ‘exposed’ area (4 cm depth at the other side). The deep area was covered by semitransparent green acrylic that protruded 10 cm from the back of the tank to provide shelter (‘cover’). We defined fish to be out of cover only when they had emerged with their full body. Our set-up reflects the ecologically relevant situation in which a fish can either rest in a safe place or explore a risky area (in search of potential food). Fish prefer to spend time under cover but, even in the absence of food, keep making regular trips out of cover to explore the exposed area (see also Harcourt et al., 2009, Jolles et al., 2014, Nakayama et al., 2013). To minimize any potential disturbances from outside the tanks, testing was conducted inside a white photo tent. The daily test order and assignment to test tanks was randomized. HD video cameras (Camileo X100, Toshiba Corporation, Japan) fixed above each tank were used to record the fish.

### Experimental Procedure

We randomly selected 156 fish from the holding tank and housed them in groups of four in the custom housing compartments. On day 1 we randomly selected one fish from each compartment (*N* = 39 focal fish) and, for visual identification, attached a small coloured plastic tag on the second dorsal spine of each fish (see Webster & Laland, 2009). To control for habituation and acclimatization effects (Biro, 2012, Gómez-Laplaza and Morgan, 2000), we allowed fish to acclimatize in their holding compartment for 2 full days. Focal fish were randomly allocated to one of three treatments and tested in the boldness test on the following 2 days (sessions 1 and 2) for 1 h per day (cf. Harcourt et al., 2009, Jolles et al., 2015). Treatment groups (*N* = 13 each) differed in their social conditions prior to the two boldness trials (see Table 1): fish were housed either (1) individually for 48 h before trial 1 as well as during the ca. 24 h period between the two trials (‘solitary’), (2) socially (i.e. with the same three fish as before) up until the first trial but individually in the ca. 24 h period between the two trials (‘partial solitary’) or (3) socially throughout (‘social’). To control for the disturbance of removing group mates, we used a fish net to unsettle the water of the solitarily housed fish compartments for 10 s. Animal care and experimental procedures were approved by the Animal Users Management Committee of the University of Cambridge as a nonregulated procedure.

### Data Analysis

Videos were tracked using custom written tracking software (by J.W.J) using Python 2.7 and the Open CV library, which was checked for any tracking errors and, if needed, manually corrected. From the tracking data we determined risk-taking behaviour as the proportion of time fish were out of cover and calculated its repeatability across the two trials. To properly determine the repeatability of risk-taking behaviour we computed three measures: (1) ‘agreement repeatability’, a measure of change in individual's trait expression across time relative to the change of the group (Lessells & Boag, 1987), using an ANOVA; (2) ‘consistency repeatability’, which measures the agreement in relative measurements between individuals (Nakagawa & Schielzeth, 2010), using an ANOVA with normalized data; and (3) rank order consistency, using robust Spearman rank correlation tests. Significance of repeatability was calculated by running 10 000 permutations of each test. To investigate temporal changes in risk-taking behaviour we fitted linear mixed models with proportion of time out of cover as the response variable, trial, treatment group and the interaction between them as fixed factors, and fish ID as a random factor. Minimal adequate models were obtained by comparing models based on log likelihood using backward stepwise elimination, starting with the full model. Residuals were visually inspected to ensure homogeneity of variance, normality of error and linearity. We used paired *t* tests to investigate whether risk taking differed between the two trials separately for each treatment group. Body size was not correlated with boldness (*P* > 0.10), in line with previous stickleback work (Bell and Sih, 2007, Jolles et al., 2015, Webster et al., 2009), and was not fitted as an additional predictor in the models. All results with *P* < 0.1 are reported as trends and *P* < 0.05 as significant. Means are quoted ± SE throughout. All data were analysed in R 3.0.2 (The R Foundation for Statistical Computing, Vienna, Austria, http://www.r-project.org).

## Results

**Figure 1:**
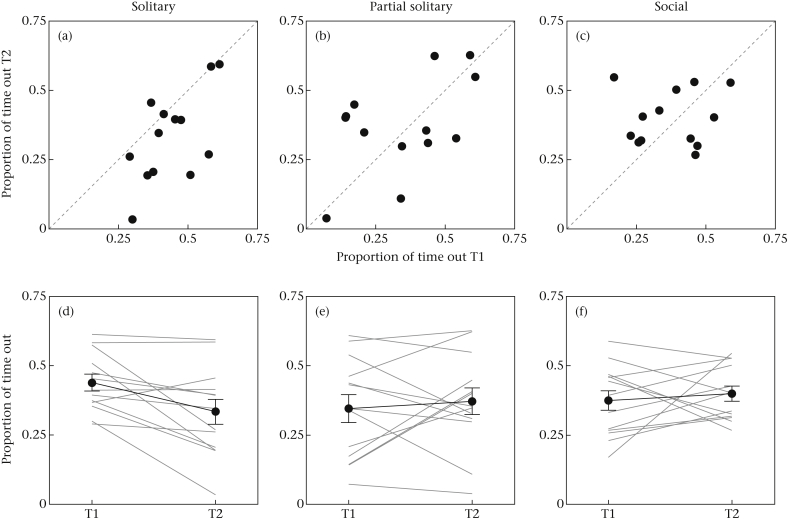
Plots showing the proportion of time that fish were out of cover during the first trial (T1) and the second trial (T2), highlighting the difference in boldness expression for fish that were housed (a, d) solitarily, (b, e) socially until the first test trial but solitarily until the second trial (partial solitary) and (c, f) socially throughout. Both (a, b, c) scatterplots and (d, e, f) line plots are presented to illustrate differences in rank order consistency, repeatability and temporal changes in risk-taking behaviour. In the line plots, grey lines depict individual responses, black lines depict group average responses, and vertical black bars depict standard errors.

On average, individuals spent 37.7% of their time out of cover. However, there was considerable interindividual variation, with some individuals only spending 3.5% of their time out of cover and others up to 62.6% during a session (Fig. 1). Overall, this individual variability in boldness was significantly repeatable across the two trials, in terms of raw consistency, consistency repeatability and agreement repeatability (Table 2).

If the social context continues to affect individual variability in behaviour even when fish are alone, then the behaviour of solitarily housed individuals should be more repeatable than that of individuals housed in a group. We found support for this hypothesis as fish housed solitarily were the only group to show significant rank order consistency and had the highest consistency repeatability, based on an ANOVA with normalized data to control for time effects (Table 2). However, in terms of agreement repeatability, there was only a nonsignificant trend for solitarily housed fish to show repeatability, while the behaviour of fish housed solitarily only the day before the second trial was repeatable. As for the other measures, fish housed socially did not show repeatability (Table 2).

Besides the differences in repeatability, the social context also affected the mean time individuals spent out of cover across both days (trial*treatment group interaction: *χ*^2^ = 6.85, *P* = 0.033): while fish housed solitarily spent significantly more time out of cover during the first than the second trial, fish from the partial social and social treatment groups did not change their behaviour significantly between trials (Fig. 1, Table 3). The three treatment groups did not differ in the total time spent out of cover in the test after pooling the data of both trials (*F*_2,36_ = 0.22, *P* = 0.805). There was no significant difference in the variance in time spent out of cover between days for all treatment groups (Table 3).

## Discussion

By keeping fish either solitarily or in a small group and subjecting them to repeated individual boldness assays, we aimed to uncover how personality expression in an individual context may be affected by prior social conditions. Although overall the behaviour of the fish was repeatable, that of fish that were housed solitarily before the personality trials was much more repeatable than that of fish housed socially, for which repeatability was not significant. Furthermore, social conditions experienced before the individual trials also affected the mean level change in boldness over time, with solitarily housed fish being bolder during the first than the second trial.

The finding that the behaviour of fish housed solitarily before the individual trials was repeatable while that of socially housed fish was not, as indicated by both rank order consistency and consistency repeatability, may potentially be explained by modulating effects of the social environment (Van den Bos et al., 2013, Webster and Ward, 2011). Previous work that compared fish in isolation versus in a group context showed that individuals take more risks (Jolles et al., 2014, Magnhagen and Bunnefeld, 2009), are more active (Gómez-Laplaza and Morgan, 1991, Webster et al., 2007) and show less attention to a moving novel object (Gómez-Laplaza & Morgan, 1986) when kept in a group, but that behavioural variability among individuals is generally higher when individuals are kept solitarily (Gómez-Laplaza and Morgan, 1986, Magnhagen and Bunnefeld, 2009). Despite being tested alone, social effects are likely to carry over to the individual test trials and compromise individual personality expression due to the recency of the social context but the effect would be much less for fish in the solitary treatment group as they had already been isolated for 2 days. Such effects may be likely, as social experiences have been shown to carry over from one social context to the next (Frost et al., 2007, Gómez-Laplaza, 2009, Jolles et al., 2014), and also after a few days of exposure to the social context, as sticklebacks may already prefer familiar individuals after 24 h (Ward et al., 2005). A second factor that is likely to have caused at least part of the difference in repeatability between the treatment groups is the effect of habituation and/or acclimatization (Bell and Peeke, 2012, Biro, 2012, Wilson et al., 1993). Only solitarily housed fish had time to acclimatize to being alone, as they would be when in the boldness environment, and individual variability in the way animals respond to changes in their environment may have compromised the behavioural repeatability of the socially housed treatment group. That is, individuals often have unique individual-specific patterns of acclimatization and habituation (Bell and Peeke, 2012, Biro, 2012). Furthermore, individuals with different personalities often have different rates of habituation, for example more easily trapped fish in the wild habituate sooner to social isolation in the laboratory (Wilson et al., 1993), and different levels of social responsiveness (Jolles et al., 2015) and plasticity of behaviour, for example shyer individuals adapt more readily to their current social environment (Jolles et al., 2014, Magnhagen and Bunnefeld, 2009). It may be hypothesized that fish experienced the removal of conspecifics as a predation threat. However, this is unlikely since the risk-taking behaviour of solitary fish was higher and not lower, as would be expected, and that of fish with the social–asocial treatment did not decrease in the second trial. As the fish in our study had relatively short exposure to their social context, approximating the relatively fluid, high-turnover groups of sticklebacks in the wild (Croft et al., 2005, Ward et al., 2002), the positive effect of social isolation on the repeatability of behaviour is most likely to be the result of the recent social context obfuscating personality expression rather than determining it. However, long-term exposure to a stable social environment may actually increase behavioural repeatability (Laskowski and Pruitt, 2014, Wolf et al., 2011). For example, a study on male water striders showed that the behaviour of individuals housed socially throughout their lives was repeatable while that of nonsocially housed individuals was not (Han & Brooks, 2014). Such effects are probably species dependent, relative to the stability of the social environment, and longer social exposure may not generate personalities in sticklebacks (Laskowski & Bell, 2014). An exciting area for future research would be to investigate how the behavioural repeatability and personality expression of adult individuals may still be affected by or even accounted for by social experiences early in life.

Had we used agreement repeatability as our personality index, we would have reached slightly different, less valid conclusions based on the low repeatability and low significance of this measure (Table 2). Despite being one of the most popular personality indices, it is often overlooked that agreement repeatability ignores any time-related change (Biro & Stamps, 2015). That is, this index looks at the change in an individual's trait expression across time relative to the change of the group (Nakagawa & Schielzeth, 2010). Although in our study the variance among individuals was the same in both trials for all treatment groups (see Table 3), complying with the first assumption of repeatability analyses (Bell et al., 2009), the significant drop in mean boldness of the solitarily housed fish would result in biased and invalid repeatability estimates. We corrected for such mean level changes by normalizing the behaviour for each group and trial, and found that, relatively, the interindividual variability in risk-taking behaviour was the most repeatable for the solitary fish. This was further confirmed by Spearman rank correlations. These analyses together highlight that it is important to consider the potential of mean level changes in behaviour in one's data, an aspect that is often ignored in personality studies (Bell et al., 2009, Biro and Stamps, 2015) beyond those that specifically focus on it (see Dingemanse et al., 2010, Dingemanse and Wolf, 2013).

The social conditions prior to the individual trials also had a temporal effect on the boldness of the fish: solitarily housed fish spent significantly more time out of cover during the first than the second trial, while no such effect was found for fish housed socially. The temporal difference in boldness between the groups is probably related to the acclimatization time to isolation (Biro, 2012). The change from the housing compartment to the solitary boldness environment was much less for fish housed solitarily already than for those housed socially until the start of the personality trial, which may have resulted in an increase in stress from social separation (see Gallup & Suarez, 1980). As a result, these fish may have been less willing to leave cover and explore the novel open environment. Previous research has suggested that isolated individuals may be more active because of their motivation to seek conspecifics, especially after recent social separation (Gallup and Suarez, 1980, Gómez-Laplaza and Morgan, 1991). However, it is unlikely that the observed higher activity of solitarily housed fish reflects social reinstatement behaviour. That is, in contrast to our study, these studies did not provide cover in their test environment, so individuals might have best reduced their predation risk by seeking others and staying close to them (Pitcher & Parrish, 1993). Other studies that used the same test set-up have shown that boldness in this task is strongly positively linked to foraging, risk-taking behaviour and leadership in a group context (Harcourt et al., 2009, Nakayama et al., 2013), highlighting that the behaviour observed during the individual test trials does not reflect social motivation. Although no robust measures of behavioural reaction norms could be acquired in the present study (see Van de Pol, 2012), visual inspection of the temporal change in behaviour (Fig. 1d, e, f) shows considerable interindividual variation, especially in the way individuals responded to the social–asocial switch (partial social treatment; Fig. 1e). An exciting avenue for future research would be to investigate behavioural reaction norms and plasticity in the way animals adapt between social and solitary environments.

Given the lack of agreement in the literature about how we should sample, design experiments and assay personality traits, it is important to evaluate our approaches and definitions (Biro, 2013, Carter et al., 2013). This study contributes to this process by showing that social (housing) conditions may affect the short-term stability of personality expression and that social isolation may improve its repeatability. Our results thus highlight that it may often be advisable to isolate individuals for a number of days prior to testing so as not to obfuscate personality expression during individual assays. However, care should be taken as many social species do not deal well with complete isolation, and long-term effects of social isolation, such as over a lifetime, may be detrimental to the repeatability of individual behaviour (Han & Brooks, 2014). Therefore the best option may be to separate individuals while allowing (some) visual and/or auditory cues of conspecifics, depending on the study system. Such result-based suggestions may be particularly relevant as studies vary considerably in the social conditions prior to individual testing, with many studies removing individuals from their social environment, either directly from the field or from their social laboratory housing (see Biro, 2012), and subsequently observe their responses during individual behavioural assays (see Webster & Ward, 2011). It is also relevant, in the context of the present study, to highlight that personality studies vary considerably in the number of times individuals are assayed. Most studies, including ours, test individuals only twice, which is generally sufficient when one is only concerned with linking a specific personality trait with another variable of interest. However, considerably more observations per animal and/or larger sample sizes may be required to get more accurate and robust repeatability estimates (Biro and Stamps, 2015, Dingemanse and Dochtermann, 2013), to rigorously characterize individual behavioural types (Biro, 2012, Biro and Stamps, 2015), and to investigate between-individual variability in plasticity and behavioural reaction norms (Dingemanse et al., 2010, Dingemanse and Wolf, 2013). An increasing number of studies actually assay individuals only once, implicitly assuming that behavioural traits are highly consistent over time (Beckmann and Biro, 2013, Garamszegi et al., 2012). Our findings highlight that such individual assays in personality research could lead to highly biased measures (cf. Beckmann and Biro, 2013, Biro, 2012, Biro and Stamps, 2015), especially when social conditions prior to testing are not considered.

To conclude, we have shown that social isolation prior to individual personality assays can improve the short-term repeatability of behaviour as recent social experiences may obfuscate personality expression. Our study adds to the increasing literature that investigates the link between animal personality and the social context (Webster & Ward, 2011), but is conceptually different from the majority of studies that have only considered social modulation effects in the social context itself. Our findings have important practical consequences for the design of personality assays, as they highlight that it is critical to consider the social conditions before such assays. Furthermore, they contribute to our understanding of the link between animal personality and the social context by emphasizing the role of carryover effects of social experiences on the stability of personality expression.

## Figures and Tables

**Table 1:** Overview of the experimental schedule

Treatment	Day 1	Day 2	Day 3	Day 4	Day 5		Day 6	
Solitary	Social	Social	*Alone*	*Alone*	T1	*Alone*	T2	End
Partial solitary	Social	Social	Social	Social	T1	*Alone*	T2	End
Social	Social	Social	Social	Social	T1	Social	T2	End

Periods when individuals were alone are shown in italics. Boldness test trials were conducted at the start of days 5 (T1) and 6 (T2).

**Table 2:** Rank order consistency, consistency repeatability and agreement repeatability of the proportion of time individuals spent out of cover across the two trials of the boldness test for each of the three treatment groups (*N* = 13 each) separately and for all fish overall

	Rank order consistency		Consistency repeatability		Agreement repeatability	
Solitary	0.61 [0.10, 0.87]	***P*=0.026**	0.64 [0.27, 1.0]	***P*=0.007**	0.38 [−0.14, 0.91]	*P*=0.081
Partial solitary	0.50 [−0.07, 0.83]	*P*=0.073	0.53 [0.09, 0.98]	***P*=0.023**	0.51 [0.05, 0.96]	***P*=0.030**
Social	0.06 [−0.50, 0.60]	*P*=0.823	0.11 [−0.50, 0.72]	*P*=0.348	0.13 [−0.47, 0.74]	*P*=0.321
Overall effect	0.37 [0.06, 0.61]	***P*=0.020**	0.38 [0.10, 0.66]	***P*=0.007**	0.38 [0.10, 0.66]	***P*=0.008**

95% confidence intervals are given in brackets and significant effects are in bold.

**Table 3:** Analyses of mean level change and variance across the two trials of the boldness test for each of the three treatment groups (*N* = 13 each) separately and for all fish overall

	Mean level change			Equal variance		
Solitary	*t*_12_=2.94	*P*=**0.012**	Yes	*F*_12,12_=0.43	*P*=0.162	Yes
Partial solitary	*t*_12_=−0.54	*P*=0.602	No	*F*_12,12_=1.11	*P*=0.854	Yes
Social	*t*_12_=−0.57	*P*=0.577	No	*F*_12,12_=1.70	*P*=0.372	Yes
Overall effect	*t*_38_=0.67	*P*=0.504	No	*F*_38,38_=0.97	*P*=0.923	Yes

Significant effect is in bold.
